# Superior clinical outcomes with combined intra‐articular platelet‐rich plasma and hyaluronic acid compared with hyaluronic acid alone in knee osteoarthritis: A prospective comparative study

**DOI:** 10.1002/jeo2.70909

**Published:** 2026-09-24

**Authors:** Trifon Totlis, Vlasiοs Achlatis, Panagiotis‐Konstantinos Emfietzis, Dimitris Tsigenopoulos, Mikail Chatzivasiliadis, Vassilis Ediaroglou, Stavros Savvakis, Efthymios Papasoulis, Ioannis Terzidis

**Affiliations:** ^1^ Thessaloniki Minimally Invasive (The‐MIS) Orthopaedic Center St. Luke's Hospital Thessaloniki Greece; ^2^ School of Medicine, Faculty of Health Sciences Aristotle University of Thessaloniki Thessaloniki Greece

**Keywords:** comparative study, hyaluronic acid, injections, knee osteoarthritis, platelet‐rich plasma, viscosupplementation

## Abstract

**Purpose:**

Although platelet‐rich plasma (PRP) and hyaluronic acid (HA) are widely used for knee osteoarthritis (KOA), it remains unclear whether combining them provides greater benefit than HA alone. This study compared the efficacy and safety of three combined injections of PRP and high molecular weight (HMW) HA to three injections of HMW HA alone in patients with KOA.

**Methods:**

A prospective comparative analysis was conducted in patients with Kellgren–Lawrence (KL) Grade II–IV KOA who underwent either three intra‐articular PRP‐HA injections (Group A) or three intra‐articular injections of HA (Group B). The Knee Injury and Osteoarthritis Outcome Score (KOOS) and subscales, the visual analogue scale (VAS) and the EuroQuality of Life–5 Dimensions–5 Levels (EQ‐5D‐5L) were assessed at baseline, 3, 6 and 12 months post‐injection. Statistical analysis was applied to compare the outcomes between the two groups. The HA used in both groups had a concentration of 1% (20 mg/2 mL) and a molecular weight of 2.8–3.2 MDa. The PRP used had been previously analysed and classified according to the Dose of injected Platelets, Efficiency of production, Purity of the PRP, Activation of the PRP (DEPA) classification as CCA.

**Results:**

The study included 150 knees from 150 patients (Group A: 75 and Group B: 75). The VAS, KOOS total and EQ‐5D‐5L scores significantly improved within each group at every time point compared to baseline (*p* < 0.05). The PRP‐HA group achieved significantly superior outcomes than the HMW‐HA group in VAS, KOOS total and EQ‐5D‐5L scores at 6‐ and 12‐month follow‐up post‐injection (*p* < 0.05). No serious adverse events were reported in either group.

**Conclusion:**

Both PRP‐HA and HA only injections significantly improved pain, function and quality of life in patients with KOA. The PRP‐HA group achieved significantly better outcomes than the HA group in all three patient‐reported outcome measures at 6‐ and 12‐month follow‐up, and in the proportion of patients who achieved Patient Acceptable Symptom State and minimum clinically important difference, especially at 12 months post‐injection.

**Level of Evidence:**

Level II.

**Abbreviations**
DEPA: Dose of injected Platelets, Efficiency of production, Purity of the PRP, Activation of the PRP
EQ‐5D‐5L: EuroQuality of Life–5 Dimensions–5 Levels
HA: hyaluronic acid
HMW: high molecular weight
IRB: Institutional Review Board
KL: Kellgren–Lawrence
KOA: knee osteoarthritis
KOOS: Knee injury and Osteoarthritis Outcome Score
MCID: minimum clinically important difference
MIBO: Minimum Information for studies evaluating Biologics in Orthopaedics
NSAIDs: non‐steroidal anti‐inflammatory drugs
OA: osteoarthritis
PASS: Patient Acceptable Symptom State
PROMs: patient‐reported outcome measures
PRP: platelet‐rich plasma
RBCs: red blood cells
VAS: visual analogue scale
WBCs: white blood cells
WOMAC: Western Ontario and McMaster Universities Osteoarthritis Index

## INTRODUCTION

Intra‐articular injections have become a common practice in the management of knee osteoarthritis (KOA), which affects approximately 23% of adults aged 40 years and older [14]. Among the available conservative treatment options, hyaluronic acid (HA) and platelet‐rich plasma (PRP) injections are two of the most widely used agents. HA improves joint lubrication and may provide anti‐inflammatory and chondroprotective effects [21, 41], while PRP contains platelets, proteins and growth factors involved in tissue repair and cartilage homoeostasis [8, 46]. Their combination has been proposed to produce synergistic effects in KOA, although the available evidence remains inconsistent [3, 17, 51].

A recent meta‐analysis even showed that PRP and HA are the most effective options in pain relief compared to other intra‐articular injection options [22]. Patients with mild‐to‐moderate KOA Kellgren–Lawrence (KL) Grades II–III respond significantly better to both intra‐articular PRP injections and HA injections individually than those with severe joint degeneration. Conversely, KL Grade IV disease represents an independent risk factor for treatment nonresponse to either PRP or HA therapy [10, 29, 39, 48].

While the individual effects of PRP and HA are well documented, comparatively fewer studies have evaluated their combined use, resulting in a limited evidence base [25]. Furthermore, the clinical response to intra‐articular therapy depends on the number of injections and compound‐specific characteristics. Although multiple PRP injections generally yield superior outcomes compared to a single injection, the optimal dosing protocol and injection interval remain subject to debate [32, 47]. Accordingly, the present study aims to contribute additional data to the existing literature and to further define the clinical role of PRP and HA combination treatment protocols in KOA. The objectives of the present study are to compare the efficacy and the safety of three combined intra‐articular injections of PRP‐HA to three intra‐articular injections of HMW HA in osteoarthritis (OA) patients' knees. It was hypothesized that the group of patients who received the combined treatment would experience superior improvements in pain relief and functional outcomes compared to those treated with HA alone.

## MATERIALS AND METHODS

### Study design

This prospective, comparative, non‐randomized controlled trial compares the efficacy of three combined intra‐articular injections of PRP + HA to three injections of high molecular‐weight HA (HMW‐HA) in patients with KOA. The patients were enroled in two centres in Thessaloniki, Greece: St. Luke's Hospital and The‐MIS Orthopaedic Centre. The study protocol was approved by the Institutional Review Board (IRB) of the Scientific Committee at St. Luke's Hospital, Thessaloniki (6 August 2021), and the study was conducted at both centres under this approval. The current study is also compliant with the principles of the Declaration of Helsinki as well as with the DEPA (Dose of injected platelets, Efficiency of production, Purity of the PRP, Activation of the PRP) and short‐MIBO (Minimum Information for studies evaluating Biologics in Orthopaedics) guidelines [33, 34]. Eligible patients were informed about the expected outcomes and potential risks of the treatment options and chose their treatment group voluntarily. Written informed consent was obtained.

Patients were then separated into two different groups depending on the treatment they received. Group A received three combined intra‐articular injections of PRP + HA, while Group B received three intra‐articular injections of HMW‐HA only. After receiving comprehensive information about both treatment options, patients were prospectively enroled and assigned to either the PRP‐HA group or the HA group through a shared decision‐making process involving the patient and the senior author. Therefore, both the patient and the primary investigator knew what treatment the patient received.

### Study population and eligibility criteria

The subjects of the study must adhere to the following criteria. Inclusion criteria were (1) patients aged 18–80 years; (2) a diagnosis of unilateral or bilateral KOA classified as Grade II, III or IV based on KL classification confirmed by radiography; (3) baseline pain intensity of four or greater on the VAS scale; (4) provision of signed informed consent prior to participation and (5) exhibited the ability to understand the instructions provided by the medical personnel.

Exclusion criteria were (1) the presence of malignancy, autoimmune disorders or immunosuppressive conditions; (2) pregnancy or breastfeeding for women of childbearing potential; (3) grade IV OA in joints other than the knee; (4) recent severe trauma to the affected knee; (5) axial knee(s) deviation >15° (varus or valgus); (6) history of allergic reactions to any components used in the treatments; (7) history of malignancy involving the musculoskeletal system; (8) signs of local or systemic infection; (9) prior intra‐articular injection in the target knee <3 months before the study; (10) surgery on the affected knee within the previous 12 months and (11) use of anticoagulants, antiplatelet, antithrombotic or nonsteroidal anti‐inflammatory drugs (NSAIDs) agents 7 days prior to study or systemic corticosteroids within 14 days of study.

### Outcome measures

The baseline data were recorded before starting treatment and included the patient demographics, relevant medical history, radiographic classifications (using standard weight‐bearing anteroposterior and lateral X‐rays to define KL grade of KOA) and initial visual analogue scale (VAS) scores.

The primary outcome measures consisted of the VAS score, the Knee Injury and Osteoarthritis Outcome Score (KOOS) along with its subscales and the EuroQol‐5D‐5L (EQ‐5D‐5L) questionnaire. These assessments were assessed at the baseline (0 months, 0 m) and repeated at 3, 6 and 12 months (3, 6 and 12 m) following the first injection. Patient‐reported outcome measures (PROMs) were obtained in person before the intervention and by telephone at each follow‐up. Minimal clinically important difference (MCID) thresholds were defined at 10 points based on Jacquet et al. for the total KOOS score (Scale 0–100), 1.4 points for the VAS score (Scale 0–10) and 0.2 points for the EQ‐5D‐5L index (Scale 0–1) [31, 51]. Additionally, a Patient Acceptable Symptom State (PASS) of 64 points for the total KOOS score and 3.0 for VAS was set [9, 31, 34].

### Intervention

#### PRP preparation

The preparation of the procedure started with the patient in a supine position, with 15 mL of venous blood drawn from the antecubital fossa using a 21 G needle in an aseptic environment. The drawn blood was processed using the ACP® double‐syringe system (Arthrex GmbH) according to the manufacturer's instructions. Anticoagulants were not used in this process. The sample was centrifuged at 1500 rpm (340 g) for five minutes at room temperature. Thereafter, approximately 4–6 mL of PRP supernatant was then transferred into the inner syringe of the double‐syringe system by pressing the wings of the double syringe together. The process of PRP production was also done under aseptic conditions [6, 50].

#### HMW‐HA preparation

Both groups received intra‐articular injections using the HMW HA OPTIVISC® viscosupplementation products (Biotech). Group A was administered OPTIVISC® HMW HA 1% (20 mg, 2.8–3.2 MDa) in a 2 mL pre‐filled syringe immediately following the PRP injection at three time points (baseline, 1 week after baseline and 2 weeks after baseline). Group B received an OPTIVISC® HMW HA 1% (20 mg, 2.8–3.2 MDa) in a 2 mL pre‐filled syringe at three time points (baseline, 1 week after baseline and 2 weeks after baseline). All syringes were individually sealed, packaged in sterile conditions and sterilized via autoclaving, ensuring sterility of both the syringe contents and their external surfaces.

### Intraarticular injection technique and post‐procedure care

The patient had to lie down in the supine position. The primary investigator performed all intra‐articular injections under strict aseptic conditions. The joint space was accessed using a lateral parapatellar approach with laterocranial angulation, after lateralization of the patella. This technique was applied across all patients in both treatment groups consistently. In Group A, PRP was administered first. Without withdrawing the needle, the PRP syringe was detached, and the syringe containing HA was then connected to the same needle hub instantly to deliver the HA into the knee joint. In Group B, only the HA syringe was administered, using the same injection technique. The patients were advised after the procedure to limit any weight‐bearing activities for 48 h and apply cold therapy to the affected knee to reduce inflammation. Patients were advised to take oral acetaminophen/paracetamol (max dose of 3 g/day, as needed) for pain management. The injection protocol was repeated weekly for 2 consecutive weeks, resulting in a total of three injections (baseline, 1 week after baseline and 2 weeks after baseline).

Both treatment groups followed the same rehabilitation protocol, which began 1 week after the injections and continued for 6 weeks. The programme consisted of progressive range of motion activities, assisted stretching and early quadriceps activation using isometric exercises. As patients progressed, the regimen was followed by closed kinetic chain exercises, resistance training for the hip and lower limb muscles, targeted balance, proprioception and gait training. Exercise intensity was altered according to the patients' pain tolerance. Gradual return to recreational activities was allowed after three weeks if the symptoms allowed for it. The PRP protocol and kit utilized in this study were identical to the standardized formulation previously characterized by Totlis et al. at our institution, classified as Grade CCA according to the DEPA system and reported in accordance with MIBO statement guidelines [24, 33, 45]. Accordingly, no additional laboratory cell‐count analysis was performed for the present patient cohort.

### Statistical analysis

The normality of distributions was explored with the Shapiro–Wilk test, skewness tests and visual representations of the distributions (histograms, boxplots, *Q*–*Q* plots, etc.). Groups were compared regarding their baseline characteristics to verify homogeneity using the independent samples *t* test, Mann–Whitney *U* test or *χ* ^2^ test. Descriptive statistics were calculated for PROMs at each group's time point. One‐way analysis of variance (ANOVA) and/or Friedman tests were conducted to assess within‐group differences over time. If they were deemed statistically significant, pairwise comparisons were followed to determine where the difference lay. To assess between‐group differences over time, the parametric repeated measures ANOVA test was conducted (along with pairwise comparisons using the Least Significant Difference correction in case of statistically significant differences). If one or more parameters were abnormally distributed at one or more of the time points, then the non‐parametric Kruskal–Wallis test was implemented to assess between‐group differences over time. Throughout the analysis, robustness checks and supplemental tests were performed when applicable.

### Sample size calculation

To estimate an acceptable sample size, the following parameters were used: a 0.05 level of statistical significance (*α* = 0.05), a *β*‐error of 20% (*β* = 0.2) resulting in a statistical power of 80% and indicative values of the main outcome measure (KOOS total) in patients with KOA before intervention (indicative mean value = 50.0), after intervention with HMW HA (indicative mean value = 60.0) and after intervention with PRP (indicative mean value = 70.0) based on the literature [16, 18]. Lastly, an indicative value of 17.0 points was considered for the standard deviation of KOOS total in patients with KOA before intervention [2, 4]. The minimum proposed sample size calculated was 46 patients for each group, assuming a 1:1 ratio, totalling 92 patients.

## RESULTS

The study included 150 knees from 150 patients (Group A: 75 and Group B: 75), which exceeded the sufficient sample size based on the study calculations. No statistically significant difference was found between groups at baseline (Table 1).

**Table 1:** Comparisons of the sample characteristics between the two groups.

	PRP + HMW HA (Group A)	HMW HA (Group B)		
Sample	Group A	Group B		
Knees	*n* = 75	*n* = 75		
Age	Group A Mean (SD)	Group B Mean (SD)	Primary comparison	Robustness check
	63.7 (10.7)	66.2 (7.2)	*p* = 0.101	*p* = 0.067
BMI	Group A Mean (SD)	Group B Mean (SD)	Primary comparison	Robustness check
	30.9 (5.9)	30.5 (3.2)	*p* = 0.658	*p* = 0.736
OA grade	Group A	Group B	Primary comparison	Robustness check
Grade II	*n* = 30	*n* = 29	*p* = 0.854	Not needed
Grade III	*n* = 26	*n* = 24		
Grade IV	*n* = 19	*n* = 22		
Parameter	Group A Mean (SD)	Group B Mean (SD)	Primary comparison	Robustness check
KOOS 0 m	51.1 (12.6)	51.0 (15.5)	*p* = 0.949	*p* = 0.776
VAS 0 m	6.2 (1.6)	6.6 (1.6)	*p* = 0.183	*p* = 0.118
EQ. 0 m	0.475 (0.232)	0.457 (0.225)	*p* = 0.628	*p* = 0.531

Abbreviations: BMI, body mass index; EQ, Euro‐Quality Of Life‐5D‐5L; HMW HA, high molecular weight hyaluronic acid; IQR, interquartile range; KOOS, Knee Osteoarthritis Outcome Score; OA, osteoarthritis; PRP, platelet‐rich plasma; SD, standard deviation; VAS, visual analogue scale.

### Post‐injection scores

**Figure 1:**
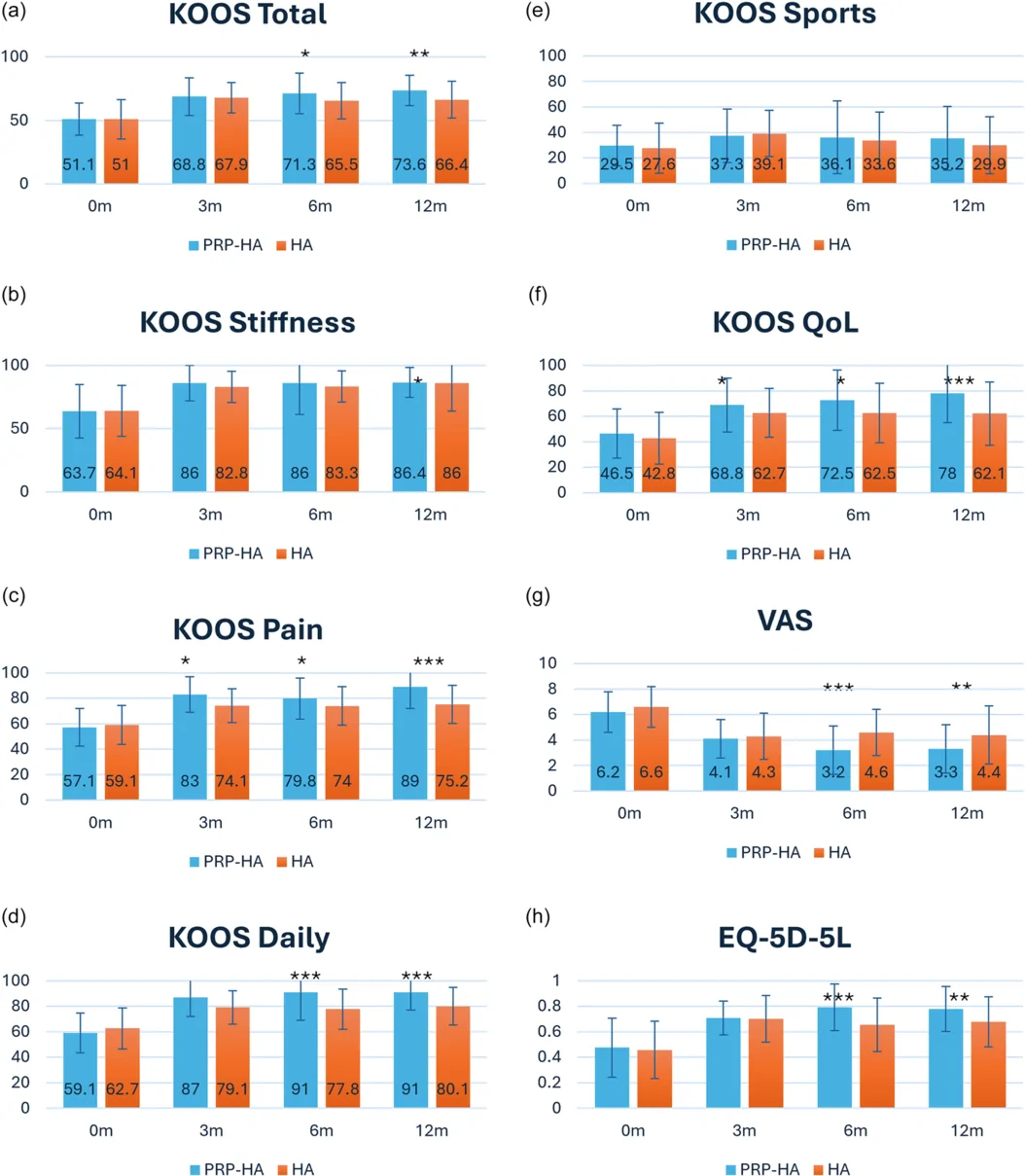
Comparison of patient‐reported outcome measures (PROMs) between the PRP‐HA and HA groups across follow‐up timepoints (baseline, 3, 6 and 12 months post‐injection). (a) Knee Injury and Osteoarthritis Outcome Score (KOOS) total, (b–f) KOOS subscales (stiffness, pain, daily living, sports/recreation, quality of life), (g) visual analogue scale (VAS) pain score and (h) EQ‐5D‐5L score. EQ‐5D‐5L, EuroQuality of Life–5 Dimensions–5 Levels; HA, hyaluronic acid; PRP, platelet‐rich plasma.

The VAS, KOOS total and EQ‐5D‐5L scores significantly improved within each group at every time point compared to baseline (*p* < 0.05). The PRP‐HA group achieved significantly better outcomes than the HMW‐HA group in VAS, KOOS total and EQ‐5D‐5L scores at 6 and 12 months follow‐up post‐injection (*p *< 0.05). Also, the PRP‐HA group showed significantly superior outcomes compared to the HMW‐HA group in KOOS stiffness, pain, daily living and quality of life KOOS subsections at 12 months post‐injection, and in pain, daily living and quality of life KOOS subsections at 6 months post‐injection (*p *< 0.05). The only KOOS subsection where no significant differences were observed between groups at all time points post‐injection was KOOS sports subsection (*p *> 0.05) (Figure 1, Table 2). Also, at 3 months post‐injection, there was no significant difference between the groups at any measured parameter (*p* > 0.05), besides the pain KOOS subsection where PRP‐HA group showed significantly better outcomes compared to HMW‐HA group (*p* < 0.05).

**Table 2:** Comparisons of PROMs' means (or medians) between the two groups at all available time points.

	PRP + HMW HA (Group A)	Within Group A comparison (compared to baseline)		HMW HA (Group B)	Within Group B comparison (compared to baseline)		Between‐groups comparison	
Parameters	Mean (SD)	Primary comparison	Robustness check	Mean (SD)	Primary comparison	Robustness check	Primary comparison	Robustness check
KOOS								
KOOS 0 m	51.1 (12.6)	N.A.	N.A.	51.0 (15.5)	N.A.	N.A.	*p* = 0.949	*p* = 0.776
KOOS 3 m	68.8 (14.9)	*p *< 0.001***	*p* < 0.001***	67.9 (11.9)	*p* < 0.001***	*p* < 0.001***	*p* = 0.699	*p* = 0.340
KOOS 6 m	71.3 (15.9)	*p* < 0.001***	*p* < 0.001***	65.5 (14.3)	*p* < 0.001***	*p* < 0.001***	*p *= 0.020*	*p *= 0.031*
KOOS 12 m	73.6 (12.0)	*p* < 0.001***	*p* < 0.001***	66.4 (14.6)	*p* < 0.001***	*p* < 0.001***	*p *= 0.001**	*p *= 0.002**
KOOS Stiffness								
KOOSstiff 0 m	63.7 (21.1)	N.A.	N.A.	64.1 (20.1)	N.A.	N.A.	*p* = 0.929	Not needed
KOOSstiff 3 m	86.0 (14.0)	*p* < 0.001***	Not needed	82.8 (12.3)	*p* < 0.001***	Not needed	*p* = 0.881	Not needed
KOOSstiff 6 m	86.0 (25.0)	*p* < 0.001***	Not needed	83.3 (12.2)	*p* < 0.001***	Not needed	*p* = 0.735	Not needed
KOOSstiff 12 m	86.4 (11.8)	*p* < 0.001***	Not needed	86.0 (22.0)	*p* < 0.001***	Not needed	*p *= 0.032*	Not needed
KOOS Pain								
KOOSpain 0 m	57.1 (14.8)	N.A.	N.A.	59.1 (15.3)	N.A.	N.A.	*p* = 0.273	Not needed
KOOSpain 3 m	83.0 (14.0)	*p* < 0.001***	Not needed	74.1 (13.2)	*p* < 0.001***	*p* < 0.001***	*p *= 0.011*	Not needed
KOOSpain 6 m	79.8 (16.1)	*p* < 0.001***	Not needed	74.0 (15.1)	*p* < 0.001***	*p* < 0.001***	*p *= 0.012*	Not needed
KOOSpain 12 m	89.0 (17.0)	*p* < 0.001***	Not needed	75.2 (15.1)	*p* < 0.001***	*p* < 0.001***	*p* < 0.001***	Not needed
KOOS Daily								
KOOSdaily 0 m	59.1 (15.6)	N.A.	N.A.	62.7 (16.2)	N.A.	N.A.	*p* = 0.089	Not needed
KOOSdaily 3 m	87.0 (15.0)	*p* < 0.001***	Not needed	79.1 (13.0)	*p* < 0.001***	*p* < 0.001***	*p* = 0.245	Not needed
KOOSdaily 6 m	91.0 (22.0)	*p* < 0.001***	Not needed	77.8 (15.8)	*p* < 0.001***	*p* < 0.001***	*p* < 0.001***	Not needed
KOOSdaily 12 m	91.0 (14.0)	*p* < 0.001***	Not needed	80.1 (14.8)	*p* < 0.001***	*p* < 0.001***	*p* < 0.001***	Not needed
KOOS Sports								
KOOSsports 0 m	29.5 (16.0)	N.A.	N.A.	27.6 (19.8)	N.A.	N.A.	*p* = 0.511	*p* = 0.250
KOOSsports 3 m	37.3 (20.9)	*p *= 0.041*	*p* < 0.001***	39.1 (18.2)	*p* < 0.001***	*p* < 0.001***	*p* = 0.574	*p* = 0.158
KOOSsports 6 m	36.1 (28.6)	*p* = 0.081	*p* = 0.056	33.6 (22.3)	*p* = 0.077	*p* < 0.001***	*p* = 0.546	*p* = 0.995
KOOSsports12 m	35.2 (25.0)	*p* = 0.134	*p *= 0.027*	29.9 (22.4)	*p* = 0.504	*p* = 0.258	*p* = 0.171	*p* = 0.447
KOOS QOL								
KOOSqol 0 m	46.5 (19.3)	N.A.	N.A.	42.8 (20.4)	N.A.	N.A.	*p* = 0.250	*p* = 0.271
KOOSqol 3 m	68.8 (21.2)	*p* < 0.001***	*p* < 0.001***	62.7 (19.1)	*p* < 0.001***	*p* < 0.001***	*p* = 0.068	*p *= 0.048*
KOOSqol 6 m	72.5 (23.7)	*p* < 0.001***	*p* < 0.001***	62.5 (23.3)	*p* < 0.001***	*p* < 0.001***	*p *= 0.010*	*p *= 0.019*
KOOSqol 12 m	78.0 (23.0)	*p* < 0.001***	*p* < 0.001***	62.1 (24.8)	*p* < 0.001***	*p* < 0.001***	*p* < 0.001***	*p* < 0.001***
VAS								
VAS 0 m	6.2 (1.6)	N.A.	N.A.	6.6 (1.6)	N.A.	N.A.	*p* = 0.183	*p* = 0.118
VAS 3 m	4.1 (1.5)	*p* < 0.001***	*p* < 0.001***	4.3 (1.8)	*p* < 0.001***	*p* < 0.001***	*p* = 0.494	*p* = 0.565
VAS 6 m	3.2 (1.9)	*p* < 0.001***	*p* < 0.001***	4.6 (1.8)	*p* < 0.001***	*p* < 0.001***	*p* < 0.001***	*p* < 0.001***
VAS 12 m	3.3 (1.9)	*p* < 0.001***	*p* < 0.001***	4.4 (2.3)	*p* < 0.001***	*p* < 0.001***	*p *= 0.002**	*p *= 0.005**
EQ‐5D‐5L								
EQ. 0 m	0.475 (0.232)	N.A.	N.A.	0.457 (0.225)	N.A.	N.A.	*p* = 0.531	Not needed
EQ. 3 m	0.709 (0.134)	*p* < 0.001***	*p* < 0.001***	0.702 (0.183)	*p *< 0.001***	Not needed	*p* = 0.220	Not needed
EQ. 6 m	0.792 (0.184)	*p* < 0.001***	*p* < 0.001***	0.655 (0.210)	*p* < 0.001***	Not needed	*p* < 0.001***	Not needed
EQ. 12 m	0.779 (0.177)	*p* < 0.001***	*p* < 0.001***	0.678 (0.196)	*p* < 0.001***	Not needed	*p *= 0.001**	Not needed

Abbreviations: EQ, Euro‐Quality of Life‐5D‐5L; HMW HA, high molecular weight hyaluronic acid; IQR, interquartile range; KOOS, Knee Osteoarthritis Outcome Score; N.A., not applicable; PROMs, patient‐reported outcome measures; PRP, platelet‐rich plasma; SD, standard deviation; VAS, visual analogue scale.

* *p* < 0.05;

** *p* < 0.01;

*** *p* < 0.001.

### MCID and PASS

A significantly higher proportion of the PRP‐HA patients' group compared with the HA group achieved PASS in VAS (56.0% vs. 28.0%, *p *< 0.001) at 6 months (Table 3) and in KOOS (74.7% vs. 57.3%, *p *= 0.025) at 12 months (Table 3). Similarly, a statistically significant higher percentage of patients undergoing PRP‐HA had MCID improvement in KOOS at 6 months (85.3% vs. 66.7%, *p *= 0.007) and at 12 months (82.7% vs. 58.7%, *p *= 0.001), in VAS at 12 months (76.0% vs. 60.0%, *p *= 0.036) and in EQ‐5D‐5L at 6 months (77.3% vs. 48.0%, *p *< 0.001) and at 12 months (66.7% vs. 40.0%, *p *= 0.001) (Table 3). There was no significant difference between the two groups, only in the proportion of patients who achieved PASS in KOOS at 6 months and VAS at 12 months, as well as in patients who reached MCID in VAS at 6 months (Table 3).

**Table 3:** Percentage of cases achieving MCID and PASS from both groups 6 months after treatment and 12 months after treatment.

	MCID		Between‐groups comparison	PASS		Between‐groups comparison
Six months after treatment	PRP‐HA	HA		PRP‐HA	HA	
KOOS	85.3%	66.7%	*p *= 0.007**	65.3%	52.0%	*p* = 0.097
VAS	78.7%	68.0%	*p* = 0.140	56.0%	28.0%	*p *< 0.001***
EQ‐5D‐5L	77.3%	48.0%	*p *< 0.001***			
Twelve months after treatment	PRP‐HA	HA		PRP‐HA	HA	
KOOS	82.7%	58.7%	*p *= 0.001**	74.7%	57.3%	*p *= 0.025*
VAS	76.0%	60.0%	*p *= 0.036*	48.0%	41.3%	*p* = 0.412
EQ‐5D‐5L	66.7%	40.0%	*p *= 0.001**			

Abbreviations: EQ‐5D‐5L, Euro‐Quality of Life‐5D‐5L; HA, hyaluronic acid; KOOS, Knee Osteoarthritis Outcome Score; MCID, minimum clinically important difference; PASS, Patient Acceptable Symptom State; PRP, platelet‐rich plasma; VAS, visual analogue scale.

* *p* < 0.05;

** *p* < 0.01;

*** *p* < 0.001.

### Adverse events

No serious adverse events were observed in either group. One patient in the PRP + HA group reported generalized muscle fatigue on the day following the intervention, which was managed with rest and resolved within 24 h. This event was not considered related to the intervention. Transient knee discomfort or pain was reported by 29 patients in the HA group and 34 patients in the PRP + HA group, with all symptoms resolving spontaneously within 1–2 days. This difference between groups was not statistically significant (*p* = 0.408). All adverse events were considered mild and required no additional intervention beyond the standard post‐injection cold therapy prescribed to all patients.

## DISCUSSION

In this prospective study, intra‐articular injections of PRP combined with HMW‐HA resulted in greater improvements in pain, function and quality of life compared with HA alone at 6 and 12 months, supported by higher rates of patients achieving MCID and PASS thresholds in the PRP‐HA group, especially at 12 months post‐injection. These findings align with and extend previous reports suggesting that PRP provides longer‐lasting symptomatic improvement than HA alone [18].

Several investigations have reported that the combination of PRP and HA yields superior clinical outcomes relative to HA monotherapy, especially beyond the early post‐injection period [20, 30, 35]. The meta‐analysis by Karasavvidis et al. also reported greater improvements in VAS at 3, 6 and 12 months and WOMAC physical function and stiffness subscores at 12 months for PRP + HA compared to HA alone [25]. Another study by Gao et al. demonstrated that the PRP + HA treatment group achieved superior clinical outcomes in midterm VAS scores compared with the HA group, exceeding the MCID threshold [20]. Similarly, the present study demonstrates the enhanced efficacy of the combined treatment. The PRP‐HA group showed significantly greater improvements than the HMW‐HA group in VAS, KOOS total and EQ‐5D‐5L scores at both 6‐ and 12‐month follow‐ups. However, at 3 months post‐injection, no significant differences were identified between groups across all PROMs and KOOS domains, except for the KOOS pain subscale. Also, not all trials have shown superiority of the PRP + HA combination against HA. A multicenter RCT reported that PRP + HA, PRP and HA had comparable efficacy at 6 and 12 months, with no significant differences in pain or function [19]. Consistent with the previous study was Zhang et al., with no clinical superiority of PRP + HA over HA at 12 months [52]. The KOOS sports and recreation subscore was the only KOOS domain without significant between‐group differences throughout follow‐up in the current study. Similar findings have been reported in a meta‐analysis comparing PRP with HA, in which no significant differences in KOOS Sports were identified across short‐, medium‐ or long‐term follow‐up despite advantages of PRP in other clinical outcomes [13]. This aligns with wider evidence showing that KOOS Sports and Recreation remains the most demanding subscale to improve and sustain, even following advanced orthobiologic injections targeting complex intra‐articular and intra‐osseous pathology in athletic cohorts [26]. Although exact treatment costs were not prospectively evaluated in the present study, prior economic analyses suggest that the higher initial acquisition cost of PRP relative to HA may be offset by its long‐term clinical benefit, though this balance remains contingent upon local healthcare setting and product pricing [37, 40].

Comparisons between PRP + HA and PRP alone have yielded conflicting results. In the analysis of Zhao et al., they found that adding HA to PRP improved VAS at 6 months and WOMAC function at 12 months without an increase in adverse events [53]. Similar results were found by Du et al. in KOA patients [15]. In contrast, Gao et al. observed no additional or sustained therapeutic advantages with PRP + HA treatment, attributing these findings potentially to the small sample size of the included trials [20]. Thus, while a synergistic effect between PRP and HA may exist, the current evidence is inconsistent and requires further validation. Moreover, emerging evidence has challenged the durability of the combination's clinical effect. Branch et al. found no long‐term advantage of PRP + HA versus PRP at 24 months [11], suggesting that any synergistic effect may be limited over time. However, Xu et al. mentioned that at 24 months, the PRP + HA group demonstrated superior pain and functional outcomes compared with the HA‐alone and PRP‐alone groups [49]. Importantly, across most trials and meta‐analyses, the safety profile of both PRP and HA has been consistent [1]. Most of the adverse events were limited to transient local reactions such as post‐injection pain, swelling or mild effusion [18, 53]. No serious adverse events such as septic arthritis, thromboembolic events or persistent joint damage have been attributed to PRP + HA in the literature [53]. The present findings are consistent with this evidence, as only minor adverse events were observed in our cohort. Current evidence suggests that PRP + HA is a safe therapeutic option for KOA with very low risks [25, 53].

The study protocol involved a series of three injections administered at weekly intervals, whereas other studies that reported more modest or inconsistent effects used only one or two injections, which may have limited the clinical benefit achieved [42, 52]. Meta‐analyses show that multiple PRP injections are associated with greater functional improvements than a single injection [32, 48]. However, the included studies employed variable dosing regimens and injection intervals. Tokyay et al. compared three PRP injections given weekly versus every 2 weeks and found no significant difference in clinical outcomes [32, 48]. Similarly, Gormeli et al. tested single, three and five weekly injections and showed greater improvements with multiple doses, but spacing itself was not the determining factor [32, 48]. Therefore, additional research is required to establish optimal dosing and injection intervals due to the heterogeneity of studies.

Studies have shown that the outcomes are more favourable in leukocyte‐poor PRP than in leukocyte‐rich PRP for knee OA [28, 36]. Some authors argue that leukocytes should be excluded from PRP formulations due to their potential pro‐inflammatory activity [38]. Although elevated platelet concentrations and higher total administered doses (absolute platelet numbers) are generally regarded as advantageous, a definitive consensus on the optimal therapeutic threshold required to promote tissue repair has yet to be established [7, 43]. The PRP in the present study was prepared with the Arthrex ACP system, which yields leukocyte‐poor PRP. As reported in a previous study conducted at the same institution by Totlis et al., the PRP was classified as CCA according to the DEPA criteria [33, 44]. In addition, the current study used an HMW‐HA, which elicits anti‐inflammatory effects and mitigates cartilage degradation compared to lower molecular weight products used in experimental models [5, 23, 27].

According to the latest European Society of Sports Traumatology, Knee Surgery and Arthroscopy–International Cartilage Regeneration & Joint Preservation Society (ESSKA–ICRS) consensus, PRP therapy is considered an appropriate intervention for individuals up to 80 years old presenting with KL Grade 0–III KOA. Nevertheless, these findings are regarded as general guidance on the various factors to consider when determining PRP use for individual patients, rather than as definitive indications [29]. Evidence regarding the use of PRP + HA in individuals with KL IV OA is limited, making it difficult to reach well‐informed conclusions. Saturveithan et al. reported that intra‐articular PRP + HA injections may serve as an alternative treatment for advanced KOA, providing functional and pain relief for up to 6 months when arthroplasty is not feasible [12].

The present study shows a positive effect of PRP + HA in patients with KL Grade II–IV KOA. The generalization of these results, though, should be made with caution and interpreted under the following limitations. The study's non‐randomized design may have led to selection bias, potentially influencing the allocation of groups. However, the sample size exceeded the number estimated during the a priori power analysis, and no significant differences were observed in baseline characteristics between the two groups. Another limitation is the inherently higher invasiveness and cost associated with the PRP‐HA treatment compared to only HA injections. Although the clinical efficacy was objectively assessed in both groups, these factors may influence patient expectations, thereby introducing potential bias. Future randomized studies with larger cohorts should investigate whether treatment response differs according to KL grade and determine whether the superior outcomes observed with PRP–HA at 12 months persist over longer follow‐up. Furthermore, establishing the optimal injection frequency and treatment interval, together with standardized PRP dosing and cost‐effectiveness evaluations, will be essential to refine the treatment protocol for combined PRP–HA therapy.

## CONCLUSION

Both PRP‐HA and HA only injections significantly improved pain, function and quality of life in patients with KOA. The PRP‐HA group achieved significantly better outcomes compared to the HA group in all three PROMs at 6 and 12‐month follow‐up, and in the proportion of patients who achieved PASS and MCID, especially at 12‐month post‐injection. No serious adverse events were reported in either group.

## AUTHOR CONTRIBUTIONS

*Conceptualization*: Trifon Totlis and Ioannis Terzidis. *Methodology*: Trifon Totlis, Vlasios Achlatis and Panagiotis‐Konstantinos Emfietzis. *Data curation*: Panagiotis‐Konstantinos Emfietzis, Dimitrios Tsigenopoulos, Vassilis Ediaroglou and Stavros Savvakis. *Formal analysis*: Vlasiοs Achlatis. *Visualization*: Trifon Totlis, Vlasios Achlatis and Dimitrios Tsigenopoulos. *Writing—original draft*: Panagiotis‐Konstantinos Emfietzis and Mikail Chatzivasiliadis. *Writing—review and editing*: Trifon Totlis and Efthymios Papasoulis. *Supervision*: Trifon Totlis, Efthymios Papasoulis and Ioannis Terzidis. All authors have read and approved the final submitted manuscript.

## FUNDING INFORMATION

The authors have no funding to report.

## CONFLICT OF INTEREST STATEMENT

The authors declare no conflicts of interest.

## ETHICS STATEMENT

The study was performed in accordance with the principles of the Helsinki Declaration and was performed in a way that always prioritized the patients' rights, safety and well‐being. The study obtained an Institutional Review Board (IRB) approval from the scientific committee of St. Luke's Hospital, Thessaloniki, on 6 August 2021 (Approval Reference: Session/Date 6 August 2021). All the patients who participated in the study have signed an Informed Consent Form (ICF).

## Data Availability

Raw research data are available upon request.
